# Editorial: Advances in Legume Research

**DOI:** 10.3389/fpls.2018.00501

**Published:** 2018-04-19

**Authors:** Diego Rubiales, Susana S. Araújo, Maria C. Vaz Patto, Nicolas Rispail, Oswaldo Valdés-López

**Affiliations:** ^1^Institute for Sustainable Agriculture, Consejo Superior de Investigaciones Científicas (CSIC), Córdoba, Spain; ^2^Instituto de Tecnologia Química e Biológica António Xavier, Universidade Nova de Lisboa (ITQB NOVA), Oeiras, Portugal; ^3^Facultad de Estudios Superiores Iztacala, Universidad Nacional Autónoma de México (UNAM), Tlalnepantla, Mexico

**Keywords:** legumes, pulses, protein crops, breeding, agronomy, domestication

In a world urgently requiring more sustainable agriculture, food security and healthier diets the demand for legume crops is on the rise. This growth is fostered by the increasing need for plant protein and for sound agricultural practices that are more adaptable and environmentally sensitive. Food, feed, fibers and even fuel are all products that come from legumes—plants that grow with low nitrogen inputs and in harsh environmental conditions.

To heighten the public awareness of the nutritional benefits of legumes as part of sustainable food production aimed toward food security and nutrition, the 68th UN General Assembly declared 2016 the International Year of Pulses. Timely, the International Legume Society (http://ils.nsseme.com) organized the Second International Legume Society Conference (ILS2) from 11 to 14th October 2016 in Portugal serving as an unique forum to present and discuss the new research accomplishments on the legume biology, as well as, seek for innovative scientific approaches intended to address these fundamental questions on this important family of plants. The health and environment benefits, as well as, the marketing of legumes were cross-cutting topics throughout the conference. As a result of contributions made to that conference, the Research Topic “Advances in legume research” (https://www.frontiersin.org/research-topics/4288/advances-in-legume-research) was designed, being also open to spontaneous submissions.

Articles published in the Research topic will be now briefly presented in this editorial introduction:

## Evolution, conservation, and diversity characterization

*Phaseolus* spp. represents an interesting model of crop evolution where five closely related species have been domesticated. Bitocchi et al. reviewed the origin of *Phaseolus* genus, the geographical distribution of the wild species, the domestication process, and the wide spread out of the center of origin. Based on this review, at least seven independent domestication events occurred in the *Phaseolus* genus. This information provides the possibility to unravel the genetic basis of the domestication process not only among species of the same genus, but also between gene pools within the same species. In particular for *P. vulgaris*, this resulted from the breaking of the spatial isolation of the Mesoamerican and Andean gene pools, which allowed spontaneous hybridization, and increasing the possibility of novel genotypes and phenotypes. Accordingly, Carović-Stanko et al. performed a genetic diversity analysis on Croatian common bean landraces and showed that about 27% were of Mesoamerican and 68% of Andean origin, while 4% of the accessions were hybrids between both gene pools.

Understanding adaptive responses to stresses is the key to genetic risk mitigation, by ensuring that crops with an appropriate assemblage of adaptive traits are grown in fitting production niches. Harnessing the genetic diversity of crop plants can be a way to achieve this goal. As a proof-of-concept, Berger et al. investigated wild and domesticated Mediterranean annual reproductive strategies comparing *Lupins* spp. collected along aridity gradients. Strong trade-offs between seed size, early vigor and phenology were observed. Despite the large differences detected for all these traits, natural and human selections have operated in very similar ways in all species.

Yellow sweet clover (*Melilotus officinalis*) is a legume species widely used as ground cover or green manure. Likewise, this legume has also the potential to be used as forage. However, this last application is not possible due to its inherent high coumarin content. To tackle this limitation, Lou et al. studied the genetic variation for herbage yield, key morphological traits, and coumarin content. Selected populations were polycrossed providing a valuable breeding pool for *M. officinalis* cultivar development in China.

## Environment and agronomy

By knowing the origin and geographic spread of a given crop it is possible to obtain clues about its environmental interactions, including relative adaptation to biotic and abiotic stresses. For instance, archaeobotanical studies have revealed that some legumes (e.g., pea, chickpea, and lentil) are considered as ancient crops of the Fertile Crescent and adjacent areas. However, it is not possible to know the history of legumes in other areas in which data are scarce, like the Bronze Age Steppes. In this respect, Dugan provided an opinion article paper suggesting that an accurate understanding of grain legumes in Proto-Indo-European and Indo-European agriculture and language will have positive consequences for our understanding of archeology, linguistics, and crop biogeography.

There is a renewed interest on intercropping due to its positive effects on crop productivity and resistance against different pathogens. In this line, Jeromela et al. reviewed and highlighted the potential of autumn- and spring-sown intercrops of annual legumes with brassicas for ruminant feeding and green manure.

Lentil producers in northern temperate regions usually apply pre-harvest desiccants as harvest aids to accelerate the lentil crop drying process and facilitate harvesting operations. Despite the beneficial effects of the different pre-harvest aids, there is little information about the impact of the whole battery of harvest aids. Subedi et al. filled this gap up by studying the effect of harvest aids alone or tank mixed with glyphosate on seed germination, seedling vigor, milling, and splitting qualities. This investigation not only confirmed the benefits of using pre-harvest aids, but also showed that the application of diquat alone or in combination with glyphosate improves lentil milling recovery yield.

## Improving responses to biotic stresses

Vicilins are seed storage proteins involved in germination processes supplying amino acids for seedling growth and plant development. Likewise, this type of proteins can have some role in plant defense. To provide more evidence about this, Jimenez-Lopez et al. studied the potential role of β-conglutins from narrow leaf lupin (*Lupinus angustifolius*) in protection against necrotrophic fungal pathogens. Transient expression of β1- and β6-conglutin proteins in *Nicotiana benthamiana* leaves demonstrated *in vivo* growth suppression of *Sclerotinia sclerotiorum* and oomycete *Phytophthora nicotianae*.

The non-host resistance is probably more durable than the single dominant resistance genes; this is because there are diverse types of effectors/elicitors and multiple resistance traits involved. The non-host resistance model with chromatin as a receptor offers flexibility to account for many plant-pathogen interactions. To prove this, Hadwiger and Tanaka studied the aspects of legume defense stimulation by salicilic acid (SA), an inducer of non-host resistance in pea tissue. Authors suggest that the SA-induced PR gene activation may be attributed to the host pea genomic DNA damage.

Heat shock transcription factors (Hsfs) are important transcription factors (TFs) in protecting plant cells from damages caused by various stresses. To increase evidence about the relevance of these TFs in legumes, Wang et al. performed a genome-wide analysis of Hsfs in *Arachis duranensis and A. ipaensis*. This analysis led to the identification of at least 16 *Hsfs* genes in these two *Arachis* species. The identified *Hsfs* genes were divided in three main groups: A, B, and C. A selection pressure analysis revealed that genes bellowing to the group B underwent a positive selection, while genes bellowing to the group A were affected by a purifying selection. Additionally, the presence of fungal elicitor responsive elements in the promoter region of some of these genes suggests their involvement in response to fungi infection.

Lectins that reside in the nucleocytoplasmic compartment are implicated in plant response to biotic and abiotic stresses. The class of Nictaba-like lectins (NLL) groups all proteins with homology to the tobacco (*Nicotiana tabacum*) lectin, known as stress-inducible lectins. Accordingly, Van Holle et al. showed that soybean NLLs are implicated in stress responses. Overexpression of two soybean NLLs homologs in Arabidopsis enhanced tolerance to bacterial infection (*Pseudomonas syringae*), insect infestation (*Myzus persicae*) and salinity.

## Improving tolerance to abiotic stresses

In field conditions, plants are exposed to multiple types of stresses. In combined stress scenario, drought can positively or negatively affect pathogen infection. To evaluate this scenario, Sinha et al. investigated the effect of drought stress on interaction of chickpea with *Pseudomonas syringae* pv. *phaseolicola* or *Ralstonia solanacearum*, and the net-effect of combined stress on chlorophyll content and cell death. This study demonstrated that, regardless of the pathogen, drought-stressed chickpea plants showed a significant reduction in the infection levels. Authors propose that both stress interaction and the net effect of combined stress could be mainly influenced by first occurring stress. Likewise, authors suggest that the outcome of the two-stress interaction in plant depends on both timing of stress occurrence and nature of infecting pathogen.

Drought is one of the major constraints limiting plant growth and yield. Modifications in the root architecture allow plants to increase their water extraction capacity and drought resistance. Another adaptation that plants use to cope with drought is an increase in the photosynthates remobilization to ensure seed production. Thus, specific shoot and root traits seem to be the key in improved resistance to drought in different crop plants. To support this, Polania et al. provide evidence indicating that the resistance to drought stress in common bean (*Phaseolus vulgaris*) is positively associated with a deeper and vigorous root system, better shoot growth, and superior mobilization of photosynthates to pod and seed production. Authors propose that pod harvest index, and seed number per area could serve as useful selection criteria for assessing sink strength and for genetic improvement of drought resistance in common bean.

Specific rooting patterns can be associated with drought avoidance mechanisms that can be used in lentil breeding programs. Idrissi et al. identified QTLs associated with root and shoot traits in a RIL of lentil. A QTL related to root-shoot ratio explained the highest phenotypic variance.

The polyamines (PAs) are low-molecular-weight organic cations found in a wide range of organisms, and perform diverse biological functions. For instance, Nahar et al. studied the physiological roles of PAs for their ability to confer salt tolerance in mung bean seedlings (*Vigna radiata*). This study revealed that exogenous PAs supplementation reduced the salt-induced oxidative stress by increasing the contents of glutathione and ascorbate as well as the activities of glyoxalase enzymes. The overall salt tolerance was reflected through improved water and chlorophyll content, as well as, seedling growth.

## Improving forage and seed quality

Improved digestibility is a main objective in forage breeding. To achieve this goal, different mapping and association approaches have been used to identify potential trait-marker that can be used for the improvement of digestibility in forage crops. For instance, Wang et al. performed an association mapping analysis in alfalfa (*Medicago sativa*) by genotyping an alfalfa panel and phenotyping for five fiber-related traits in four different environments. From this study, eight associations were predicted in two environments, whereas 20 markers were predicted to be associated with multiple traits. The identification of these trait-marker associations will help to breed alfalfa cultivars with high forage quality.

Quinolizidine alkaloids are toxic secondary metabolites found within the genus *Lupinus*. While they offer the plants protection against insect pests, their accumulation in grains complicates its use for food purposes as high levels confer a bitter taste and may result in acute anticholinergic toxicity. In this line, Frick et al. discuss possibilities for further elucidation and manipulation of the quinolizidine alkaloids pathway in lupin crops by using conventional and cutting-edge technologies.

Seed-coat cracking and undesirable seed coat color highly affect external appearance and commercial value of peanuts (*Arachis hypogaea*). To face this issue, Wan et al. performed a whole-genome transcriptome analysis on a peanut mutant with cracking and brown colored seed coat (*pscb*). This analysis led to the identification of three candidate genes for the trait, which can be used as marker genes for plant breeding.

High germination, nutritional quality, and yield potential under high heat and dryland production conditions are priority seed traits in soybean (*Glycine max*). Bellaloui et al. searched for these traits in exotic germplasm and identified three breeding lines with consistently superior germination. The study also unveiled potential roles of minerals, especially K, Ca, B, Cu, and Mo, in maintaining high seed quality.

Starch phosphorylase (PHO) catalyses the reversible transfer of glucosyl units from glucose-1-phosphate to the non-reducing ends of α-1,4-D-glucan chains with the release of phosphate. Qin et al. identified three PHO isoform (*LjPHO*) genes in the *Lotus japonicus* genome. Overexpression studies suggested that *LjPHO3* may participate in transitory starch metabolism in *L. japonicus* leaves, but its catalytic properties remain to be studied.

## Improving plant nutrition

Legumes establish root symbioses with rhizobia that provide plants with nitrogen (N) through biological N fixation (BNF), as well as with arbuscular mycorrhizal (AM) fungi that mediate improved plant phosphorus (P) uptake. Püschel et al. studied the interplay between BNF and AM symbioses in *Medicago truncatula* and *M. sativa* along a P-fertilization gradient. The AM symbiosis generally improved P uptake by plants and considerably stimulated the efficiency of BNF under low P availability. In contrast, under high P availability the AM symbiosis brought no further benefits to the plants. Results also suggested competition for limited C resource between the two microsymbionts. The use of P-efficient genotypes is a sustainable management strategy for enhancing yield and P use efficiency. Zhou et al. identified genetic variation for P use efficiency in soybean genotypes under field conditions and studied hydroponically P assimilation characteristics and the related mechanisms of P-efficient soybean genotypes.

Iron deficiency is a major problem in many countries raising interest in biofortification of legumes. Tan et al. reviewed the current status of iron biofortification discussing challenges and potential application of transgenic technology.

Multiple genes and TFs are involved in the uptake and translocation of iron in plants from soil. Sen Gupta et al. developed molecular markers for iron metabolism related genes using genome synteny with *M. truncatula*.

## Understanding plant physiology

Plant hemoglobins (Hbs) are found in nodules of legumes and actinorhizal plants but also in non-symbiotic organs of monocots and dicots. Non-symbiotic Hbs (nsHbs) have been classified into two phylogenetic groups. Class 1 nsHbs show an extremely high O_2_ affinity and are induced by hypoxia and nitric oxide (NO), whereas class 2 nsHbs have moderate O_2_ affinity and are induced by cold and cytokinins. Using spectroscopic analyses, Calvo-Begueria et al. showed major differences between the two phylogenetic classes of nsHbs and also between the two members of the same class, strongly suggesting that these three globins perform non-redundant functions.

Photoperiod is one of the major environmental factors determining time to flower initiation and first flower appearance in plants. Daba et al. studied photoperiod sensitivity in chickpea (*Cicer arietinum*). Photoperiod-sensitive and -insensitive phases were identified by experiments in which individual plants were reciprocally transferred in a time series from long to short days and vice versa in growth chambers. Results from this research will help to develop cultivars with shorter pre-inductive photoperiod-insensitive and photoperiod-sensitive phases to fit to regions with short growing seasons.

Light is essential for plant growth and development. Yuan et al. studied the response of cultivated lentil and wild *Lens* germplasm to different light environments, showing that days to flower of *Lens* genotypes were mainly influenced by changes in the red/far-red ratio of the light quality but not by changes in the intensity of the photosynthetically active radiation. The distinctly different responses between flowering time and elongation under low red/far-red conditions among wild *Lens* genotypes suggest that flowering and elongation are controls by discrete pathways. Three *L. lamottei* and one *L. ervoides* genotypes were less sensitive to changes in light quality maintaining similar yield, biomass, and harvest index across all three light environments; these are indications of better adaptability toward changes in light environment.

The phytohormone auxin plays also a critical role in regulation of plant growth and development as well as in plant responses to abiotic stresses. Auxin transporters are major players in polar auxin transport. Chai et al. performed a genome-wide analysis of the soybean *GmLAX* auxin transporter gene family. A total of 15 *GmLAX* genes were identified in the soybean genome distributed on 10 out of the 20 soybean chromosomes. *GmLAX*s showed very dynamic expression patterns, most of them responsive to drought, salt and dehydration stresses, as well as, auxin and abscisic acid stimuli, in a tissue- and/or time sensitive manner.

MADS-domain proteins are important TFs involved in many aspects of plant reproductive development. Chi et al. found that *GmAGL1* is specifically expressed in reproductive tissues but not in roots, stems, and leaves of soybean. The ectopic overexpression of *GmAGL1* in Arabidopsis suggested a role for this MADS-box protein in floral organ identity and fruit dehiscence.

Excessive flower and pod abscission represents an economic drawback for yellow lupine (*Lupinus luteus*). Glazinska et al. studied transcriptional networks in the pods, flowers and flower pedicels to identify genes playing key roles in generative organ abscission in yellow lupine. Auxin, ethylene and gibberellins were some of the main factors engaged in generative organ abscission. Identified differentially expressed genes common for all library comparisons were involved in cell wall functioning, protein metabolism and water homeostasis and stress response.

Improvement of seed quality requires deep insights into the genetic regulation of seed development. The endosperm serves as a temporary source of nutrients that are transported from maternal to filial tissues. It also generates signals for proper embryo formation. Zhang et al. showed that soybean *GmZOU-1* gene is an ortholog of the Arabidopsis bHLH domain TF that may be responsible for endosperm breakdown and embryo cuticle formation in soybean.

## Adjusting plant growth and development

Plant morphology markedly affects its competitive ability and persistence in mixtures. Faverjon et al. compared the patterns of shoot organogenesis and shoot organ growth in contrasting forage species belonging to the four morphogenetic groups (i.e., stolon-formers, rhizome-formers, crown-formers tolerant to defoliation and crown-formers intolerant to defoliation). The consequences of this quantitative framework are discussed, along with its possible applications regarding plant phenotyping and modeling.

Seed dispersal and germination are two key traits that have been selected to facilitate cultivation and harvesting of crops. Hradilová et al. studied anatomical structure of seed coat and pod, and identified metabolic compounds associated with water-impermeable seed coat and differentially expressed genes involved in pea (*Pisum* spp.) seed dormancy and pod dehiscence. This integrated analysis of seed coat in wild and cultivated pea provides new insight as well as raises new questions associated with domestication, seed dormancy and pod dehiscence.

Determinacy growth habit and accelerated flowering are adaptive traits in common bean. Through a comparative mapping approach, González et al. detected additive and epistatic QTLs regulating flowering time, vegetative growth, and rate of plant production. Further QTL analysis coupled with previous results highlighted *001G189200* gene, homologous to the *Arabidopsis thaliana TFL1* gene, as a candidate gene for determinacy locus.

Mungbean (*Vigna radiata*) is a cleistogamous plant in which flowers are pollinated before they open, which prevents yield improvements through heterosis. Chasmogamous mutant (CM) plants are available. Chen et al. mapped the *cha* gene responsible for the chasmogamous flower trait to a 277.1-kb segment on chromosome 6. Twelve candidate genes were detected in this segment, including *Vradi06g12650*, which encodes a YUCCA family protein associated with floral development. A single base pair deletion producing a frame-shift mutation and a premature stop codon in *Vradi06g12650* was detected only in CM plants suggesting *Vradi06g12650* as *cha* candidate gene.

The time of flowering has a major influence in both plant productivity and adaptation to the changing environment. Hence, different efforts to understand the genic programs underlying the flowering time in important crop plants have been made. For instance, Srivastava et al. used a high-throughput multiple QTL-seq strategy to identify two major QTL genomic regions governing flowering time on chickpea (*Cicer arietinum*) chromosome 4. The functionally relevant molecular tags delineated can be used for deciphering the natural allelic diversity-based domestication pattern of flowering time and expediting genomics-aided crop improvement to develop early flowering cultivars of chickpea.

The branching habit is an important descriptive and agronomic character of peanut (*Arachis hypogaea*). Kayam et al. fine-mapped this trait by combining high-throughput sequencing and bulk segregant analysis, providing a baseline for candidate gene discovery and map-based cloning.

Proper phenotyping is becoming a bottleneck in breeding, being particularly difficult for inherent root system architectures. To overcome this constrain, Zhao et al. provided machine learning algorithms that were used for unbiased identification of most distinguishing root traits and subsequent pairwise pea (*Pisum sativum*) cultivar differentiation.

Fine mapping of quantitative trait loci (QTL) and qualitative trait genes plays an important role in gene cloning, molecular-marker-assisted selection (MAS), and trait improvement. As a proof-of-concept, Li et al. mapped 26 agronomic QTLs and five qualitative trait genes related to pigmentation in adzuki bean (*Vigna angularis*). For this mapping analysis, authors used 1,571 polymorphic SNP markers generated via Restriction-site-Associated DNA sequencing technology. The identification of these QTL and qualitative trait genes will contribute to breed adzuki bean cultivars with desirables traits.

Another example about the relevance of the QTL analysis in plant breeding programs is provided by Srivastava et al. who used a high-throughput whole genome next-generation resequencing strategy to develop InDel markers in chickpea (*Cicer arietinum*) mapping populations. By using this approach, Srivastava et al. identified three major QTLs governing pod number and seed yield per plant. These functionally relevant molecular tags can drive marker-assisted genetic enhancement to develop high-yielding cultivars with increased seed/pod number and yield in chickpea.

## Genomic tools for breeding

Genetic variation is the basis for plant breeding programs. Most conventional crop improvement programs rely on natural genetic variation present among germplasm pools. Alternatively, induced mutagenesis still offers the potential to create valuable genetic variation for genetic enhancement and breeding. For instance, Horn et al. identified agronomically desirable cowpea (*Vigna unguiculata*) mutants after gamma irradiation. Ten phenotypically and agronomically stable novel mutants are described that constitute a valuable genetic resource for cowpea genetic enhancement and breeding.

The large-scale mining and high-throughput genotyping of novel gene-based allelic variants in natural mapping population are essential for association mapping to identify functionally relevant molecular tags governing useful agronomic traits. For instance, Bajaj et al. used an EcoTILLING approach coupled with agarose gel detection assay to discover 1133 novel SNP allelic variants by genotyping in a *desi* and *kabuli* chickpea collection constituting a seed weight association panel. Integrating genotyping and phenotyping data identified eight SNP alleles in the eight TF genes regulating seed weight of chickpea.

Ištvánek et al. used illumina paired-end sequencing of red clover (*Trifolium pratense*) allowing the identification of large sets of SSRs and SNPs throughout that will be key for implementing genome-based breeding approaches, for identifying genes underlying key traits, and for genome-wide association studies.

Genotyping-by-Sequencing (GBS) may drastically reduce genotyping costs compared with SNP array platforms. Annicchiarico et al. compared GBS protocols on legume species that differ for genome size, ploidy, and breeding system, and showed successful applications and challenges of GBS data on legume species. Authors devised a simple method for comparing phenotypic vs. genomic selection in terms of predicted yield gain per year for same evaluation costs, whose application to preliminary data for alfalfa and pea in a hypothetical selection scenario for each crop indicated a distinct advantage of genomic selection.

## Author contributions

All authors listed have made a substantial, direct and intellectual contribution to the work, and approved it for publication.

### Conflict of interest statement

The authors declare that the research was conducted in the absence of any commercial or financial relationships that could be construed as a potential conflict of interest.

